# Functional Roles of Arbuscular Mycorrhizal Fungi and Plant Growth-Promoting Rhizobacteria in Pistachio: Implications for Stress Tolerance, Nutrient Acquisition and Disease Suppression

**DOI:** 10.3390/plants15172575

**Published:** 2026-08-24

**Authors:** Luis Vera, Jorge Retamal-Salgado, Gonzalo Tortella, Gustavo Santoyo, Mauricio Schoebitz

**Affiliations:** 1 Facultad de Agronomía, Universidad de Concepción, Víctor Lamas 1290, Concepción 4070386, Chile; lvera2025@udec.cl; 2 Instituto de Investigaciones Agropecuarias, INIA Quilamapu, Chillán 3800062, Chile; jorge.retamal@inia.cl; 3 Centro de Excelencia en Investigación Biotecnológica Aplicada al Medio Ambiente (CIBAMA), Facultad de Ingeniería y Ciencias, Universidad de La Frontera, Temuco 4811230, Chile; gonzalo.tortella@ufrontera.cl; 4 Institute of Biological and Chemical Research, Universidad Michoacana de San Nicolás de Hidalgo, Morelia 58030, Mexico; gustavo.santoyo@umich.mx; 5 Centro de Biotecnología, Universidad de Concepción, Barrio Universitario s/n, Concepción 4070386, Chile; 6 Soil and Ecosystem Functions Research Center of Chile (CISFECh), Universidad Austral de Chile, Valdivia 5090000, Chile

**Keywords:** *P. vera*, arbuscular mycorrhizal fungi, plant growth-promoting rhizobacteria, abiotic stress tolerance, nutrient acquisition

## Abstract

Pistachio (*Pistacia vera* L.) is among the most economically important nut crops worldwide. They are increasingly exposed to the environmental constraints associated with climate change, including drought, salinity, nutritional imbalances, and heightened disease pressure. These stressors compromise plant growth, physiological performance, nutrient acquisition, and orchard productivity, highlighting the need for sustainable strategies to enhance crop resilience. This review critically examines the current knowledge on the functional roles of arbuscular mycorrhizal fungi (AMF) and plant growth-promoting rhizobacteria (PGPR) in pistachio production. Evidence indicates that AMF and PGPR contribute to plant performance through multiple complementary mechanisms, including improved nutrient mobilization and uptake, maintenance of ionic homeostasis, enhancement of water-use efficiency, stimulation of antioxidant defenses, modulation of stress-related signaling pathways, and suppression of phytopathogens. AMF primarily enhance phosphorus acquisition, water relations, and soil structural stability, whereas PGPR contribute to nutrient solubilization, biological control, and induction of plant defense responses. Despite promising experimental results, most studies have been conducted under controlled conditions, limiting the translation of microbial inoculation strategies to commercial orchards in the field. We identified the key knowledge gaps and research priorities required to improve the consistency, scalability, and field validation of microbiome-based approaches for sustainable pistachio production under increasingly challenging environmental conditions.

## 1. Introduction

Pistachio (*Pistacia vera* L.) is a species native to the region between Afghanistan and northern Iran [1]. During the Middle Ages, this crop spread throughout the Mediterranean Basin. In the late 19th century, *P. vera* was introduced to America and Australia [2]. *P. vera* is cultivated for commercial use. Other species, such as *P. atlantica*, *P. terebinthus*, *P. khinjuk*, and *P. lentiscus*, are also used as rootstocks [3]. Pistachios are of great economic importance [4]. In 2024, global pistachio production reached 1.4 million tons (3.1 billion pounds). The leading producers are the United States, Turkey, and Iran, which together account for 87% of the global production [5]. Beyond overall production volumes, the major producing regions differ markedly in edaphic constraints, which may, in turn, shape the performance of microbial inoculants. In California, the leading producer, orchards are largely established on alluvial soils of marine origin in the San Joaquin Valley that are naturally prone to salinization, whereas in Iran’s Rafsanjan province—historically a major global supplier—cultivation relies on calcareous, saline-alkaline soils increasingly affected by groundwater depletion; both regions have experienced measurable production declines linked to soil salinization [6]. Comparative evidence on how these contrasting soil environments shape AMF and PGPR efficacy remain limited, but recent evidence from calcareous soils in Türkiye indicates that PGPR-mediated benefits are strongly genotype-dependent and diminish once salinity exceeds moderate levels, with no protective effect detected under severe stress [7]. This suggests that microbial inoculant strategies developed under one regional soil context cannot be assumed to transfer directly to other calcareous or saline pistachio-growing environments without local validation.

*P. vera* adapts well to saline soils and prolonged drought periods. Therefore, it is cultivated in various semi-arid regions worldwide [8]. In addition, pistachios offer numerous health benefits. From a nutritional standpoint, they are notable for their high protein, fiber, monounsaturated fat, minerals, vitamins, bioactive compounds (carotenoids, phenolic acids, flavonoids, and anthocyanins), and polyphenols (which have antioxidant and anti-inflammatory properties for humans) [9,10].

Pistachio cultivation faces growing constraints due to climate change and intensifying extreme weather events, including reduced winter chill units, rising summer temperatures, high relative humidity, and increased solar radiation [10,11]. These conditions have affected walnuts, causing dehiscence, embryonic abortion, and increased susceptibility to abiotic stressors under extreme conditions, as well as fungal diseases affecting leaves and roots [12]. In Mediterranean and semi-arid regions, drought, salinity, and extreme temperatures disrupt water status, ionic homeostasis, and key physiological processes in plants, negatively affecting crop growth and yields [13,14].

Rising temperatures can shorten the pollen-receptive period of most female varieties and advance the flowering of some male cultivars, thereby increasing phenological imbalances in pistachio trees [15]. These limitations are further exacerbated in saline or calcareous soils, as reduced nutrient availability, such as phosphorus (P) and zinc (Zn), hinders optimal crop development [16,17]. High relative humidity favors foliar diseases, whereas waterlogging induces root hypoxia and increases susceptibility to root and collar rot [11,18].

Increasing evidence indicates that beneficial rhizosphere microorganisms play a central role in plant adaptation to environmental stresses. Plant growth-promoting rhizobacteria (PGPR) and arbuscular mycorrhizal fungi (AMF) contribute to plant performance through multiple, often complementary, mechanisms at the root–soil interface. Under abiotic stress conditions, PGPR can enhance plant tolerance by modulating abscisic acid (ABA) signaling, producing volatile organic compounds (VOCs), and expressing ACC deaminase activity, while also promoting osmotic adjustment and antioxidant defense [19,20]. Likewise, AMF improve water and nutrient acquisition through the expansion of extraradical hyphal networks and regulation of aquaporin activity, thereby contributing to the maintenance of plant water status under adverse environmental conditions. In addition to their role in abiotic stress tolerance, both microbial groups can contribute to plant health under biotic stress [20]. AMF may limit pathogen establishment through competition for ecological niches and resources, whereas PGPR can suppress pathogens through the production of inhibitory metabolites and other mechanisms of microbial antagonism [21].

In *P. vera*, a crop of major economic importance cultivated primarily in arid and Mediterranean environments, resilience to environmental stress depends not only on plant physiological adaptations but also on the interactions occurring at the root–soil interface [22,23,24]. Within this framework, the rhizosphere microbiome represents a potentially important yet underexplored component of pistachio adaptation to adverse environmental conditions. Studies conducted in *P. vera* and related rootstocks have reported positive responses to AMF or PGPR inoculation under specific experimental conditions. Nevertheless, the evidence is still limited, heterogeneous, and largely restricted to greenhouse and nursery trials, preventing firm conclusions regarding the consistency of these effects under commercial orchard conditions [25,26]. Moreover, differences among microbial inoculants, rootstocks, environmental conditions, and experimental approaches have hindered the development of a coherent understanding of their agronomic relevance in pistachio production systems. Consequently, the consistency of these responses under commercial orchard conditions remains poorly understood, limiting the validation and large-scale implementation of microbiome-based strategies for sustainable pistachio production under increasing environmental pressures. This review summarizes the main biotic and abiotic stress factors affecting the growth of *P. vera*. Within this framework, we critically examine the available evidence on the physiological, biochemical, and microbiological mechanisms through which PGPR and AMF can enhance stress tolerance in pistachio trees. It also identifies the main experimental limitations, knowledge gaps, and translation barriers that currently restrict the validation and field-level application of these microbial strategies.

Although many of the mechanisms discussed in this review have been characterized in crop species other than *P. vera*, they are used to provide the mechanistic framework for interpreting plant–microorganism interactions. Whenever available, evidence obtained directly from pistachio is highlighted, whereas findings from other species are clearly identified as supporting information to discuss potential mechanisms relevant to pistachio cultivation. AMF may contribute to the suppression of aboveground diseases through systemic mechanisms extending beyond the root system. Mycorrhizal colonization has been associated with mycorrhiza-induced resistance (MIR), which involves the priming of plant immune responses and enhanced activation of jasmonic acid- and salicylic acid-related defense pathways following pathogen challenge [27,28]. Such systemic responses could contribute to limiting diseases caused by foliar and canker-associated pathogens affecting pistachio, including *Septoria* spp., *Neoscytalidium* spp., and *Botryosphaeria* spp. PGPR may similarly contribute to disease suppression beyond local antagonistic interactions in the rhizosphere through systemic modulation of plant defense responses. Furthermore, AMF–PGPR co-inoculation has been associated with a threefold increase in mycorrhizal intensity and the abundance of arbuscules and vesicles, together with a 1.5-fold increase in mycorrhizal frequency and enhanced activation of plant defense responses against fungal phytopathogens [29]. Beyond disease suppression, these beneficial plant–microbe interactions may also contribute to stress tolerance through improved nutrient acquisition, maintenance of ionic homeostasis and plant water status, osmotic adjustment, antioxidant protection, and modulation of stress-responsive signaling pathways.

### Literature Search and Synthesis Strategy

This review was a structured narrative synthesis of the available literature on interactions among *P. vera*, arbuscular mycorrhizal fungi (AMF), and plant growth-promoting rhizobacteria (PGPR) under biotic and abiotic stress conditions. The literature was retrieved primarily from the Web of Science Core Collection, Scopus, and Google Scholar databases. Additional relevant publications were identified through the reference lists of selected articles. The literature search was conducted between January and May 2026 and included publications available up to May 2026.

The literature search included combinations of keywords such as *P. vera*, pistachio, arbuscular mycorrhizal fungi, AMF, plant growth-promoting rhizobacteria, PGPR, drought, salinity, nutrient acquisition, biocontrol, disease suppression, abiotic stress, and microbial inoculation. Boolean operators (“AND” and “OR”) were used to refine the searches.

Peer-reviewed original research articles constituted the primary source of evidence. Review articles were used to provide conceptual background and to identify additional primary studies. Particular emphasis was placed on studies involving Pistacia species. However, relevant mechanistic studies from other crop species were included when they provided broadly accepted evidence supporting AMF- or PGPR-mediated physiological, biochemical, or microbiological mechanisms applicable to pistachio. Pure in vitro microbial studies without plant evaluation were generally excluded unless they provided fundamental mechanistic information directly related to microbial functions discussed in this review.

The literature screened was critically evaluated and synthesized according to the major functional roles of AMF and PGPR in nutrient acquisition, stress tolerance, and disease suppression in pistachio. This review is therefore intended as a structured narrative synthesis of the available evidence rather than a formal systematic review.

## 2. *P. vera* Under Stress

**Figure 1:**
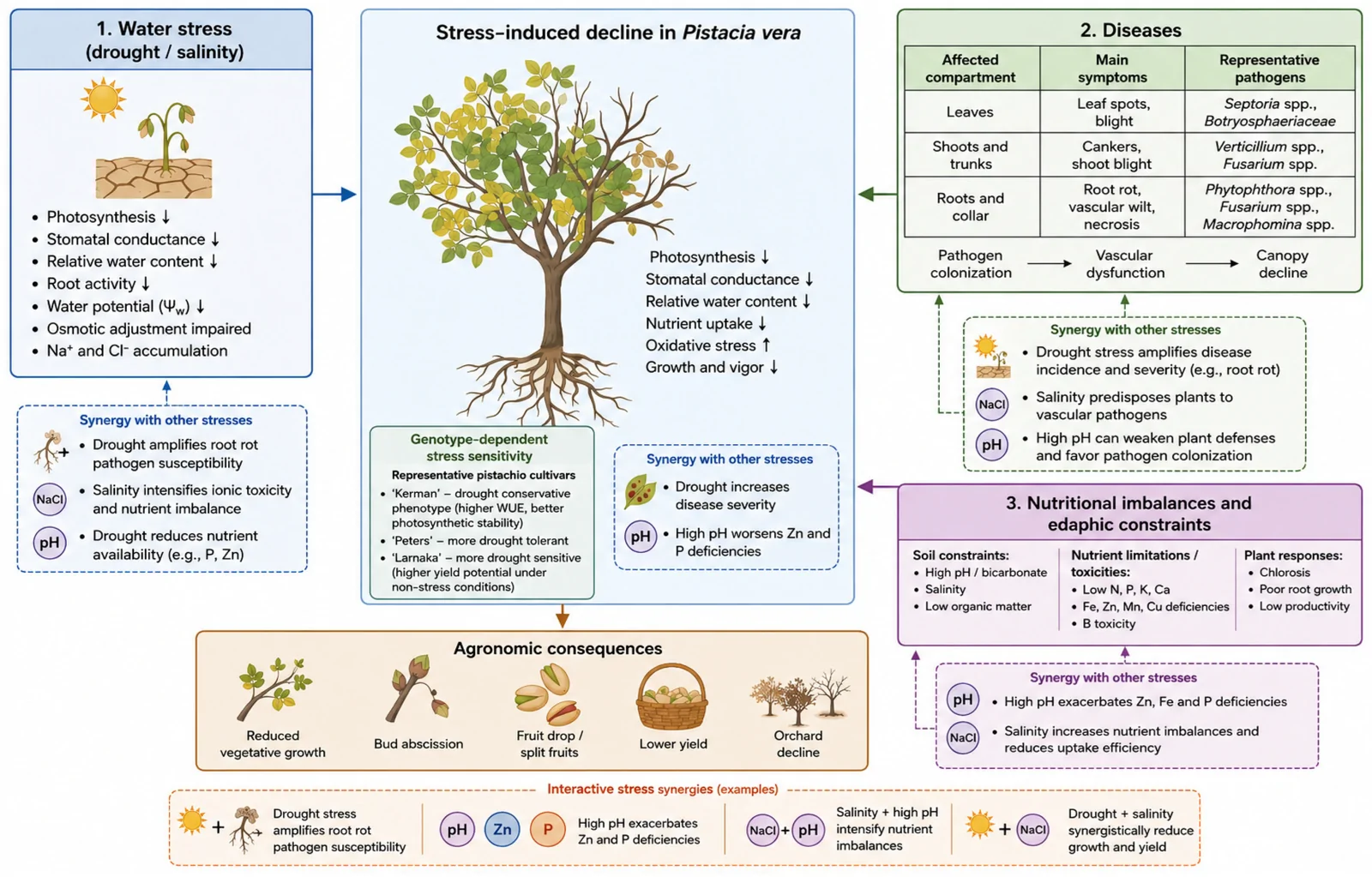
Major constraints affecting *P. vera* under drought, salinity, disease pressure, and nutritional imbalances. The schematic illustrates the main environmental and biological stressors affecting pistachio production and their physiological consequences. Water stress reduces photosynthesis, stomatal conductance, relative water content (RWC), root activity, and water potential (Ψw), while impairing osmotic adjustment and promoting Na^+^ and Cl^−^ accumulation. Biotic stress caused by foliar, vascular, and soil-borne pathogens compromises plant health through tissue damage, vascular dysfunction, and root necrosis. Nutritional limitations associated with alkaline or calcareous soils, salinity, low organic matter, and nutrient deficiencies further restrict plant growth and productivity. Together, these stressors disrupt photosynthesis, nutrient uptake, and water relations, leading to oxidative stress, reduced vegetative growth, bud abscission, fruit drop or fruit splitting, lower yield, and orchard decline. RWC, relative water content; Ψw, water potential. Created in Biorender. Tortella (2026) https://BioRender.com/1lgugzw.

Drought, salinity, nutritional imbalances, and disease pressure are among the main constraints threatening pistachio production across most of the world’s major pistachio-growing regions [30]. These limitations are expected to intensify under climate change scenarios, as increasing temperatures and altered precipitation patterns may exacerbate water scarcity, soil salinization, pathogen pressure, and physiological stress in Mediterranean and semi-arid orchards [31]. Under biotic and abiotic stress conditions, pistachio trees experience disruption of hormonal homeostasis, which directly affects bud retention. A reduction in auxin transport from developing buds, combined with increased ethylene and abscisic acid (ABA) accumulation, enhances the sensitivity of abscission zones and promotes bud drop [32]. In parallel, decreased levels of cytokinins and gibberellins further compromise bud growth and maintenance, ultimately leading to reduced tree vigor and increased bud abscission [33]. Therefore, bud drop should not be interpreted as an isolated reproductive disorder, but rather as an integrated response to environmental stress, tree vitality, and the balance between carbon allocation, water status, nutrient supply, and defense metabolism. Understanding how these factors interact is essential for identifying the physiological and ecological processes that ultimately determine pistachio productivity and resilience under adverse environmental conditions (Figure 1).

### 2.1. Nutrient Imbalance

Nutritional imbalances represent a major constraint for *P. vera*, particularly in arid and semi-arid environments, where soil alkalinity, salinity, carbonate accumulation, boron toxicity, and low organic matter commonly restrict nutrient availability (Figure 1). Nutrient uptake in pistachios is phenologically regulated and generally peaks during the nut-filling stage, although environmental conditions can modify this pattern and influence nutrient demand [34]. Severe environmental stress and nutrient deprivation can disrupt reproductive development, contributing to flower and fruit abscission, and ultimately reducing yield [35]. In this context, climate change can alter the relationship between yield fluctuations and variations in the mineral nutrient composition of pistachio tree shoots and leaves, thereby affecting flower abscission [36]. Deficiencies of N, P, K, and Ca have been reported in commercial orchards, while N and Mn deficiencies can strongly reduce leaf chlorophyll content and, consequently, photosynthetic performance [37,38].

Similarly, micronutrient deficiencies under high pH and carbonate-rich conditions may contribute to pre-harvest fruit abscission, yield reduction, and alternate bearing [20]. In calcareous and saline-sodic soils, P fixation is particularly problematic because it limits root development, growth rates, and biomass allocation [17]. Therefore, nutritional disorders in pistachios should not only be understood as isolated nutrient deficiencies but also as the outcome of complex interactions among soil chemistry, climate variability, phenology, reproductive demand, and orchard management practices.

### 2.2. Disease Pressure

Biotic stress in *P. vera* is strongly influenced by interactions among host defense mechanisms, drought intensity, pathogen pressure, and environmental conditions (Figure 1). Although pistachios activate transcriptomic pathways related to jasmonic acid (JA) and salicylic acid (SA), which are involved in abiotic stress tolerance and pathogen defense, these mechanisms may not fully prevent disease development when trees are exposed to prolonged drought, high temperatures, or poor soil conditions [39,40]. Under water stress conditions, the most common diseases in the Mediterranean Basin are panicle and shoot rot caused by *Botryosphaeria* and leaf spot caused by Septoria [18]. Septoria leaf spot is a significant foliar disease that causes severe defoliation and yield reduction and is prevalent in major pistachio-growing regions [41].

Climate change may further intensify these problems by favoring aggressive canker-forming pathogens such as *Neoscytalidium dimidiatum* and *N. mesopotamica*, which have been reported to cause extensive cankers in pistachio seedlings [42]. In addition to foliar and woody tissue diseases, belowground pathogens pose a major threat by directly compromising root function, water acquisition, and vascular integrity of the host. *Phytophthora* spp. can cause root and crown rot, rapid decline, and tree death at different developmental stages [43].

Other soil-borne pathogens, including *Fusarium solani*, *Macrophomina phaseolina*, and *Verticillium dahliae*, have been associated with root necrosis, stem discoloration, vascular dysfunction, and decline symptoms in pistachios and related rootstocks [44,45,46]. Bacterial pathogens, such as *Pantoea agglomerans* and *Xylella fastidiosa*, also pose significant phytosanitary concerns, as they can induce necrotic symptoms or leaf scorch by colonizing the vascular system, blocking xylem flow, and impairing water movement within the plant [47,48]. Insects may contribute additional stress, as aphid-induced galls alter the morphology, anatomy, and histochemistry of pistachio leaves during development [49]. Overall, the available evidence suggests that pistachio diseases and pest-related damage should be analyzed within an integrated framework linking drought stress, vascular dysfunction, root health, canopy physiology, and management, as these factors frequently interact to determine disease severity, tree performance, and long-term orchard productivity (Figure 1).

### 2.3. Drought Constraints

Drought stress alters key physiological processes in *P. vera*, particularly gas exchange, plant water relations, root function, and reproductive performance (Figure 1). Recent evidence shows that drought reduces net photosynthesis (Pn), stomatal conductance, transpiration rate, and mesophyll efficiency across different photosynthetic photon flux densities [50]. At the whole-plant level, drought-stressed pistachio plants exhibit reduced photosynthetic pigment content, lower relative water content, weaker root development, limited osmotic adjustment, and impaired water status [51]. These physiological disruptions ultimately translate into reduced growth, lower vigor, and diminished reproductive performance, making drought one of the most critical constraints affecting pistachio productivity in arid and semi-arid regions (Figure 1).

The negative effects of water stress caused by salinity, as observed in pistachio plants, include reduced biomass and relative water content (RWC), as well as increased O_2_•^−^, H_2_O_2_, malondialdehyde (MDA), and electrolyte leakage [52]. Salinity stress altered morphological traits and reduced nutrient content, relative water content (RWC), and membrane stability index (MSI). In contrast, it increased the accumulation of K, Ca, Na, glycine betaine, reactive oxygen species (ROS), and malondialdehyde (MDA), indicating ionic imbalance, oxidative stress, and membrane damage [53]. Under field conditions, restricted soil water availability can reduce stem water potential (Ψs) and affect fruit development, including an increased incidence of split fruits [54]. Interestingly, Pn appears to be less sensitive to water deficit than stomatal conductance, suggesting that stomatal closure acts as an early drought-avoidance mechanism before severe impairment of carbon assimilation occurs [55]. Drought stress alters the relative water content and carbohydrate metabolism of the Iranian pistachio cultivars Akbari, Kaleghochi, and Ohadi, as reflected by changes in soluble sugar levels and starch accumulation [56]. Furthermore, it significantly affects the growth and nutrient absorption of pistachio seedlings [57]. These responses indicate that drought affects not only plant water status but also carbon allocation, nutrient acquisition, and metabolic homeostasis, thereby influencing the overall plant performance (Figure 1).

The cultivars ‘Golden Hills’, ‘Lost Hills’, and ‘Kerman’ differ in stomatal length, width, area, and density, indicating that stomatal anatomy may contribute to the contrasting drought-response strategies among pistachio genotypes [58]. In this context, ‘Kerman’ showed the lowest Ψs, leaf stomatal conductance (gs), and Pn values. However, the highest intrinsic water-use efficiency (WUEi) supports the idea that this cultivar follows a conservative water-use strategy based on tighter stomatal regulation [59]. Overall, the available evidence suggests that drought tolerance in pistachios is a multifaceted trait involving coordinated adjustments in water relations, photosynthetic performance, carbon metabolism, nutrient acquisition, and stomatal regulation, all of which ultimately influence tree productivity and resilience under water-limited conditions (Figure 1).

## 3. Arbuscular Mycorrhizal Fungi: Nutritional and Stress Modulators

**Figure 2:**
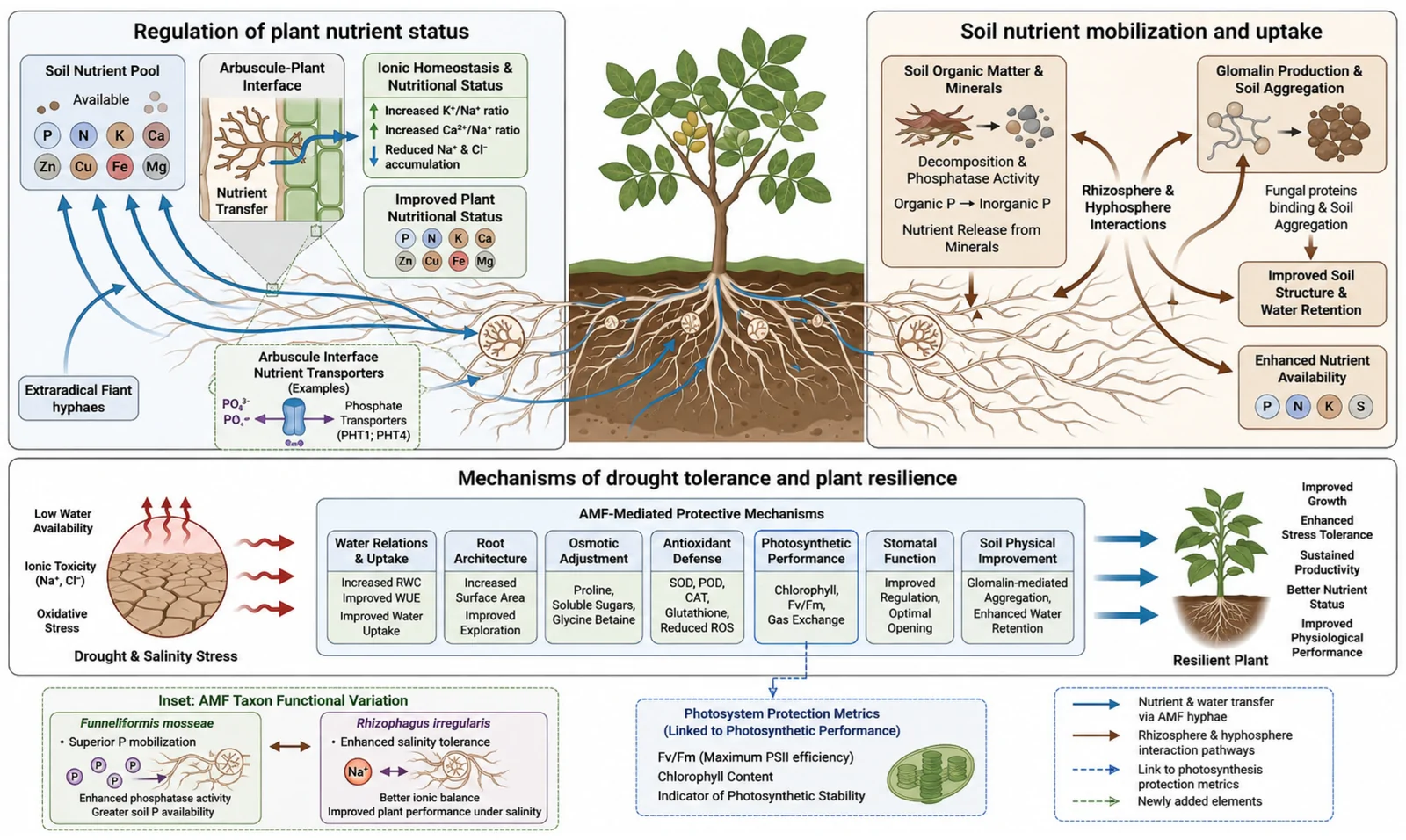
Functional mechanisms through which arbuscular mycorrhizal fungi (AMF) enhance plant performance under abiotic stress conditions in *P. vera*. The upper-left panel illustrates the role of extraradical hyphae in improving nutrient acquisition at the arbuscule–plant interface, thereby increasing the uptake of essential macro- and micronutrients and maintaining ionic homeostasis. The upper right panel summarizes AMF’s contribution to soil nutrient mobilization through organic matter decomposition, phosphatase activity, rhizosphere and hyphosphere interactions, glomalin production, soil aggregation, and improved nutrient availability. The lower panel illustrates the coordinated physiological mechanisms underlying drought and salinity tolerance, including improved water relations, enhanced root architecture, osmotic adjustment, antioxidant defense, maintenance of photosynthetic performance, regulation of stomatal function, and improvements in soil physical properties. Together, these mechanisms increase plant resilience, resulting in improved growth, stress tolerance, nutrient status, and productivity under adverse environmental conditions. RWC, relative water content; WUE, water-use efficiency; SOD, superoxide dismutase; POD, peroxidase; CAT, catalase; ROS, reactive oxygen species; Fv/Fm, maximum quantum efficiency of photosystem II. Created in Biorender. Tortella (2026) https://BioRender.com/rhufgkr.

AMF constitute a unique group of obligate root symbionts that establish mutualistic associations with approximately 90% of terrestrial plant species, serving as a key link between plants and soil mineral nutrients [60]. Their effects on plant growth and ecological stability are highly context-dependent and vary according to plant genotype, fungal taxon, soil properties, and environmental conditions [61,62,63]. As a result, AMF are increasingly recognized not only for their role in plant nutrition but also for their contribution to plant adaptation and resilience under adverse environmental conditions, such as drought. This variability is particularly relevant for mitigating abiotic stress, as AMF-mediated responses can enhance plant tolerance to drought, salinity, and nutrient deficiencies [64]. Through their extensive hyphal networks and intimate association with plant roots, AMF influence nutrient acquisition, water relations, soil structure, and stress-responsive physiological processes, thereby contributing to the stability and function of the plant–soil system (Figure 2).

### 3.1. Regulation of Plant Nutrient Status

AMF symbiosis is regulated by the nutritional status of the host plant, allowing plants to balance the costs and benefits of mycorrhizal colonization [65]. In mycorrhizal roots, nutrient uptake occurs through two complementary pathways: the direct root pathway and the mycorrhizal pathway, the latter being mediated by extraradical AMF hyphae and nutrient transfer in arbuscule-containing cortical cells [66]. These hyphal networks extend soil exploration beyond the root depletion zone and enhance access to poorly mobile nutrients, particularly phosphorus [67].

The extraradical hyphae of AMF effectively increase the absorptive surface area of the root system, enabling the acquisition of nutrients that would otherwise remain inaccessible through root uptake. Consequently, instead of exclusively relying on the direct root pathway, plants activate an alternative mycorrhizal absorption pathway [68,69]. One well-studied mechanism is the uptake of inorganic phosphate by the extraradicular mycelium of AMF, its translocation to intraradicular structures, and its subsequent release into the peri-arbuscular space. Plant- and fungus-specific transport proteins mediate the symbiotic acquisition of nutrients within this interface [70]. Phosphorus-mediated regulation of AMF symbiosis is accompanied by the systemic control of strigolactone production [71]. Recent transcriptomic studies have further demonstrated that AMF colonization induces coordinated regulation of genes involved in nutrient transport, hormone signaling, and stress adaptation [60,65]. Differential expression of phosphate transporters, aquaporins, transcription factors, and genes associated with ABA- and jasmonate-mediated signaling indicates that AMF symbiosis promotes extensive molecular reprogramming beyond nutrient acquisition alone [65]. These responses highlight that successful mycorrhizal establishment depends on a complex regulatory network integrating nutritional, physiological, and defense-related pathways. Therefore, in some cases, it increases the absorption and accumulation of essential macronutrients, including P, K, Ca, and Mg in plants [72].

In Pistacia, the combined application of AMF with organic amendments, such as vermicompost or compost, can significantly enhance phosphorus uptake compared with that in non-inoculated plants [57]. In general, AMF inoculation improves plant nutritional status by promoting the uptake and accumulation of essential elements, particularly P and Zn, as well as K, Ca, Mg, N, Mn, and Cu in roots and leaves [73,74]. Under saline conditions, these benefits are often associated with improved ionic homeostasis, as reflected by higher K^+^/Na^+^ and Ca^2+^/Na^+^ ratios and reduced Na^+^ and Cl^−^ accumulation [75]. Similarly, under salt stress, AMF inoculation has been shown to increase shoot and root dry weights, enhance P and Fe accumulation, and improve ionic balance [53]. In addition to nutrient acquisition, AMF can influence nutrient partitioning within plant tissues. Leaf elemental analyses have shown the selective accumulation of Ca, Fe, Mg, N, and S in mycorrhizal plants [76]. This effect is particularly relevant under stress conditions, where AMF help maintain nutrient balance by improving the acquisition of nutrients with low mobility or limited availability, such as P and Zn, through extensive extraradical hyphal networks [77]. Mycorrhizal pistachio plants showed considerably higher mineral nutrient contents, including P, N, K, Ca, Zn, and Cu, than non-mycorrhizal plants, both under adequate irrigation and water stress conditions [78]. Collectively, these findings indicate that AMF contribute to plant nutrition by enhancing nutrient uptake and improving nutrient-use efficiency, ionic homeostasis, and nutrient distribution within plant tissues, particularly under environmental stress conditions (Table 1; Figure 2).

An additional factor contributing to AMF performance variability is the pistachio rootstock genotype. Commercial pistachio production relies predominantly on grafted trees, and rootstocks such as *Pistacia atlantica*, *P. terebinthus*, *P. integerrima*, and their hybrids differ in root architecture, nutrient uptake capacity, and tolerance to drought and salinity. These characteristics may influence AMF colonization efficiency, carbon allocation to the fungal symbiont, and the magnitude of plant nutritional and physiological responses following inoculation. Although arbuscular mycorrhizal colonization has been documented across major pistachio rootstocks, differences in rootstock physiology may affect the functional outcomes of these symbioses under field conditions [79]. Consequently, rootstock genotype likely represents an important source of variability in the effectiveness of AMF inoculation and should be considered when selecting microbial inoculants for commercial orchards. Future research should further investigate rootstock × microorganism interactions to optimize the establishment and long-term performance of beneficial microbial symbioses under diverse environmental conditions.

### 3.2. Soil Nutrient Mobilization and Uptake

Inoculation with arbuscular mycorrhizal fungi can improve growth, nutrient uptake, and root colonization in species of the genus Pistacia. [80]. Host plants recognize AMF-derived signaling molecules, including lipochitooligosaccharides and chitooligosaccharides, via LysM receptor-like kinases. This recognition triggers the common symbiosis signaling pathway, facilitating fungal accommodation, arbuscule development, and efficient nutrient exchange between the two symbiotic partners [80]. In particular, AMF symbiosis has been associated with increased plant growth and enhanced P absorption [57]. This response may be partly explained by increased acid phosphatase activity in the mycorrhizosphere, which facilitates phosphorus acquisition from soil compartments otherwise inaccessible to roots [81]. Through this mechanism, AMF can contribute to the solubilization and mobilization of organic phosphorus in soil, thereby improving its availability to plants (Table 1; Figure 2).

Beyond their direct effects on plant nutrition, AMF play an important role in regulating soil nutrient dynamics and biogeochemical processes that influence nutrient availability in the rhizosphere. Overall, AMF represent a biological strategy for enhancing soil functionality and plant nutrition through symbiotic associations with plant roots. These associations promote nutrient uptake, improve soil structure, and mitigate the effects of abiotic stress. In addition, AMF can contribute to carbon sequestration by producing glomalin-related soil proteins that enhance soil aggregate stability [82]. AMF inoculation has also been shown to increase the availability of soil macronutrients, including N, P, K, and S, as well as their uptake and accumulation in roots and shoots [83]. Emerging evidence suggests that different AMF groups differ in their ability to mobilize soil organic matter and exchange nutrients with host plants, likely due to differences in their life-history strategies [84]. AMF can accelerate leaf litter mineralization by increasing saprophytic biomass and enzymatic activity, including that of nitrate reductase, urease, and cellulase. In turn, saprophytic microorganisms may enhance AMF colonization, thereby expanding the zones of high enzymatic activity in the soil [85]. AMF also contribute to soil quality by producing organic acids and glomalin-related compounds, which can reduce erosion, chelate heavy metals, promote carbon sequestration, and maintain soil macroaggregation. In addition, they can stimulate phosphatase activity, particularly alkaline phosphatase, thereby increasing soil phosphorus availability [86]. AMF inoculation may further stimulate key nutrient-cycling processes by increasing the activity of N-cycling enzymes, including β-N-acetylglucosaminidase (NAG) and leucine aminopeptidase (LAP), and P-cycling enzymes, such as alkaline phosphatase (ALP) and acid phosphatase (ACP). These enzymatic changes may be accompanied by increases in total nitrogen, total phosphorus, ammonium-N (NH_4_^+^-N), nitrate-N (NO_3_^−^-N), and available phosphorus [87]. Collectively, these findings indicate that AMF influence nutrient acquisition not only through direct nutrient transfer to plants but also by modifying soil biochemical processes, microbial interactions, and nutrient cycling pathways that regulate soil fertility and ecosystem function (Table 1; Figure 2).

### 3.3. Mechanisms of Drought Tolerance and Plant Resilience

The AMF-mediated improvement of plant tolerance to abiotic stress is widely recognized, positioning these symbionts as a promising and environmentally friendly strategy to enhance crop productivity while reducing the ecological impacts associated with conventional agricultural inputs [88]. AMF establish symbiotic associations with plant roots and modulate drought-induced physiological, biochemical, and molecular responses [75,89]. Beyond their nutritional role, AMF function as key regulators of plant stress physiology, coordinating multiple mechanisms involved in drought adaptation and plant resilience. Under water deficit, AMF symbiosis can mitigate stress-induced impairment of the photosynthetic apparatus by preserving chlorophyll content and sustaining higher photosynthetic rates and photochemical efficiency [75]. These responses are often accompanied by improved plant growth, increased root colonization, and favorable morphological adjustments associated with stress tolerance. In parallel, AMF enhance physiological protection by promoting glycine betaine accumulation, maintaining higher relative water content (RWC), improving membrane stability, and reducing ionic and oxidative stress under salinity conditions. These responses are reflected by lower Na^+^ and Cl^−^ accumulation in leaves and roots, increased membrane stability index (MSI), and reduced levels of oxidative stress markers such as MDA and H_2_O_2_ [53,73].

AMF-mediated tolerance to water stress involves integrating morphological, hydraulic, biochemical, and edaphic mechanisms. At the root level, AMF promote a dual adaptive strategy based on root morphological plasticity and symbiotic assistance, increasing root length, surface area, and architectural complexity (Table 1; Figure 2). Simultaneously, the extraradical hyphal network expands the volume of soil explored for water and nutrient uptake [90]. These morphological responses are complemented by improved hydraulic regulation, including the modulation of aquaporin expression and water transport, which contributes to plant water homeostasis under drought conditions [91]. At the biochemical level, AMF promote osmotic adjustment by accumulating compatible solutes, such as proline and soluble sugars, while helping maintain photosynthetic activity under water deficit (Figure 1)**.** Simultaneously, AMF enhance antioxidant defenses by activating enzymatic and non-enzymatic ROS-scavenging systems in both plant and fungal partners, thereby reducing oxidative damage and improving stress acclimation [92]. These responses are further integrated with ABA- and MAPK-mediated signaling pathways, osmolyte metabolism, aquaporin regulation, and ion homeostasis through transporters such as SOS1 and NHX under saline conditions [93]. At the soil level, AMF release glomalin-related soil proteins that enhance soil aggregation, pore stability, and water retention. In contrast, mycorrhizal networks can contribute to water uptake and hydraulic redistribution, particularly in perennial systems [94,95]. Together, these mechanisms support both immediate drought tolerance and long-term resilience of plant–soil systems. Several of these mechanisms have also been documented in Pistacia species, suggesting that the drought-protective functions attributed to AMF are directly relevant to pistachio cultivation under water-limited conditions (Table 1; Figure 2).

In pistachio seedlings, AMF inoculation alleviated salinity-induced damage by significantly improving the relative RWC, root surface area, and root morphology. Moreover, mycorrhizal seedlings exhibited lower proline accumulation, which was associated with reduced oxidative stress and lower MDA concentrations, likely mediated by enhanced antioxidant defense mechanisms [52]. In addition, AMF can improve photosynthetic performance under stress conditions by promoting more efficient electron flow, reducing energy dissipation, and enhancing productive photosynthetic activity at different points along the photosynthetic electron transport chain. In this context, the maximum quantum yield of PSII photochemistry (Fv/Fm) is frequently used as an indicator of photoinhibition or other stress-induced damage to PSII [96]. Under mild and moderate water stress, mycorrhizal pistachio plants showed higher relative chlorophyll and carotenoid contents, along with greater gas exchange capacity [97]. Similarly, under greenhouse salinity conditions, AMF inoculation improved leaf RWC, root surface area, and root morphology, thereby increasing plant biomass and salinity tolerance. These responses were accompanied by enhanced activity of antioxidant enzymes, including SOD, POD, CAT, and GR, as well as increased levels of non-enzymatic antioxidants, such as reduced glutathione (GSH), oxidized glutathione (GSSG), α-tocopherol, carotenoids, and phenolic compounds, in pistachio seedlings exposed to salinity stress [52]. Studies on pistachio confirm that AMF-mediated protection against drought and salinity involves coordinated improvements in plant water status, root development, photosynthetic performance, osmotic adjustment, and redox regulation. Mycorrhizal seedlings generally maintained higher relative water content, biomass, chlorophyll content, gas exchange, and PSII efficiency, together with lower electrolyte leakage and concentrations of MDA and H_2_O_2_. These responses were accompanied by changes in compatible solutes and antioxidant metabolism, although the magnitude and direction of individual biochemical responses varied among AMF taxa, pistachio genotypes, and stress conditions [52,75]. Overall, the available evidence indicates that AMF enhance pistachio resilience by maintaining physiological function rather than by uniformly activating a single antioxidant or osmoprotective pathway.

Despite the generally positive effects reported for AMF, the magnitude of plant responses varies considerably among studies. This variability largely reflects differences in fungal identity, host genotype and rootstock, soil physicochemical characteristics, water deficit intensity, and experimental conditions. Different AMF taxa differ in colonization efficiency, extraradical hyphal development, nutrient exchange capacity, and regulation of plant physiological responses, resulting in distinct functional outcomes. Likewise, pistachio genotypes may differ in their responsiveness to mycorrhizal colonization because of differences in root architecture, carbon allocation, and symbiotic compatibility. Therefore, contrasting effects reported across studies should not be interpreted as contradictory evidence but rather as context-dependent responses determined by multiple interacting biological and environmental factors.

Collectively, these findings indicate that AMF-mediated stress tolerance in *P. vera* is driven by the integration of improved water and nutrient acquisition, maintenance of photosynthetic performance, osmotic adjustment, ionic balance, antioxidant defense, and soil structural improvement (Table 1; Figure 2), highlighting the multifunctional role of AMF in enhancing plant resilience under drought and salinity stress.

**Table 1:** Functional traits and plant responses associated with AMF inoculation in *P. vera* under abiotic and biotic stress conditions.

Functional Component	AMF Taxon/Inoculum	Stress Condition	Inoculant Dosage	Pistachio Cultivar/Plant Material	Specific AMF-Mediated Response or Trait	Ref.
**Regulation of plant nutrient status**	*Claroideoglomus etunicatum* (currently *Entrophospora etunicata)*	Drought stress (50% of well-watered irrigation)	100 g AM inoculum pot^−1^ (10–12 spores g^−1^ dry soil; ~65% root infection potential)	*P. vera* cv. Badami seedlings	Improved shoot and root biomass, leaf area, chlorophyll content, nutrient uptake (P, N, K, Ca, Zn and Cu), osmotic adjustment (soluble sugars and proline), peroxidase activity, and drought tolerance.	[78]
	*Rhizophagus irregularis*	Salinity stress (250 mM NaCl)	100 g inoculum pot^−1^ (10–12 spores g^−1^ dry soil; 60–70% root infection potential)	*P. vera* L. cv. Ohadi seedlings	Improved plant growth, nutrient uptake (especially P and Fe), K^+^/Na^+^ and Ca^2+^/Na^+^ ratios, relative water content (RWC), membrane stability index (MSI), glycine betaine accumulation, and regulation of ion transporter genes (SOS1, SKOR, CCX2, NRT2.4, PIP2.4, and PHO1) involved in ionic homeostasis and salinity tolerance.	[53]
	*Funneliformis mosseae + Rhizophagus intraradices + Funneliformis caledonius* (AMF mixture), separately or in combination with *Trichoderma* spp.	Root-knot nematode (*Meloidogyne javanica*)	60 g inoculum pot^−1^, applied 3 months before inoculation with Trichoderma; nematodo: 5000 J2 pot^−1^	*P. vera* cv. Badami Zarand	Increased shoot, leaf and root biomass; enhanced uptake of P, K, Fe, Zn and Cu; reduced nematode damage, supporting improved plant growth and nutritional status.	[74]
	*Funneliformis mosseae*	Drought (40–100% field capacity) and salinity (0.91–24.63 dS m^−1^ NaCl)	200 g inoculum pot^−1^ (≈800 spores g^−1^ inoculum; ~90% infected roots)	Four pistachio rootstocks (*P. vera* ‘Badami-Riz-e-Zarand’, ‘Qazvini’, ‘Sarakhs’, and UCB1 hybrid)	Increased shoot and root biomass, relative water content, chlorophyll, and uptake of N, P, K, Mg, Ca, Fe, and Zn; reduced Na^+^ and Cl^−^ accumulation under salinity; decreased H_2_O_2_, MDA, and methylglyoxal, improving oxidative balance and abiotic stress tolerance.	[73]
**Soil nutrient mobilization and uptake**	Native AMF community (*Glomus* spp., *Funneliformis* spp., and unidentified *Glomus* spp.)	Drought stress (well-watered, moderate, severe)	100 g AMF inoculum pot^−1^ (≈120 spores g^−1^ inoculum); compost or vermicompost at 4% w/w	*P. vera* cv. Badami-Rize-Zarand	Improved root colonization, shoot and root biomass, and significantly enhanced P and Zn uptake under drought. Vermicompost increased root colonization, whereas compost combined with AMF produced the greatest improvements in nutrient uptake under drought stress.	[57]
	*Funneliformis mosseae*	Salinity stress (EC 12 dS m^−1^ NaCl)	100 g inoculum pot^−1^ (10–15 spores g^−1^ dry soil; 71% root infection potential)	*P. vera* L. cv. Akbari seedlings	Enhanced uptake of P, K, Ca and Mg, improved K^+^/Na^+^ and Ca^2+^/Na^+^ ionic homeostasis, and increased nutrient acquisition efficiency, contributing to improved mineral nutrition under salinity stress.	[75]
**Mechanisms of drought tolerance and plant resilience**	*Funneliformis mosseae, Rhizophagus* intraradices and *Funneliformis mosseae*	Drought stress (25–100% field capacity) and salinity stress (0.5–15 dS m^−1^)	100 g inoculum pot^−1^ (≈75–90% colonized inoculum)	*P. vera* cvs. Badami-Riz-Zarand, Qazvini, Sarakhs, Abareqi, and Banebaqi *(P. eurycarpa × P. mutica)*	Enhanced photosynthetic performance through improved PSII photochemistry (Fv/Fm and PIABS), electron transport efficiency, gas exchange, chlorophyll and carotenoid contents, while reducing energy dissipation under drought and salinity stress, thereby increasing plant resilience to abiotic stress.	[96,97]
	*Funneliformis mosseae*	Salinity stress (EC 12 dS m^−1^ NaCl)	100 g inoculum pot^−1^ (10–15 spores g^−1^ dry soil; 71% root infection potential)	*P. vera* L. cv. Akbari seedlings	Improved photosynthetic performance (Fv/Fm and gas exchange), water-use efficiency (WUE), osmotic adjustment (glycine betaine and soluble sugars), antioxidant capacity (α-tocopherol and carotenoids), and ionic homeostasis, while reducing MDA, H_2_O_2_, and electrolyte leakage, thereby enhancing salinity tolerance and plant resilience.	[75]
	*Claroideoglomus etunicatum (currently Entrophospora etunica-ta* and *Claroideoglomus etunicatum*	Drought stress	100 g inoculum pot^−1^	*P. vera* cv. Akbari seedling	Improved drought tolerance by increasing biomass, chlorophyll content, nutrient acquisition (P, K, Zn, Cu), osmotic adjustment (soluble sugars and proline), flavonoid accumulation, and peroxidase activity, thereby enhancing plant growth and stress resilience.	[98]
	*Rhizophagus irregularis*	Salinity (300 mM NaCl)	100 g inoculum pot^−1^	*P. vera* cv. Kaleghochi seedlings	Enhanced salinity tolerance by improving biomass, root morphology and leaf relative water content, reducing ROS (O_2_•^−^, H_2_O_2_), MDA and electrolyte leakage, increasing enzymatic (SOD, CAT, POD, GR) and non-enzymatic antioxidants (ascorbate, carotenoids and α-tocopherol), and modulating antioxidant gene expression (Cu/Zn-SOD, Fe-SOD, Mn-SOD, CAT, POD and GR).	[52]

Note: Arbuscular mycorrhizal fungal nomenclature has been updated according to the currently accepted taxonomy. Species names reported under the former genus Glomus in the original publications are presented here using their currently accepted names.

## 4. PGPR: Multifunctional Root Allies

### 4.1. Nutrient Solubilization and Uptake

Plants maintain nutrient homeostasis through the coordinated regulation of nutrient uptake, transport, and storage, enabling metabolic adjustments to fluctuations in soil nutrient availability and internal tissue demand [99]. At the root–soil interface, nutrient acquisition is shaped by dynamic interactions among root activity, soil physicochemical properties, and resident microbial communities [100]. Root exudates generate chemically heterogeneous microenvironments that influence microbial recruitment and colonization patterns, thereby modifying nutrient transformation and availability in the rhizosphere [25]. Within this framework, PGPR may contribute to nutrient acquisition by mobilizing N, P, K, and micronutrients through mechanisms that depend on soil pH, mineral composition, root exudation, and microbial persistence [101]. Therefore, their functional relevance is expected to be greater in soils where nutrient availability is constrained by salinity, alkalinity, drought, or low fertility. However, these effects must be interpreted in the context of specific plant–soil–microbe systems [102]. In addition, PGPR may influence nutrient acquisition indirectly by modifying root architecture, stimulating root hair development, and altering rhizosphere chemistry through the production of phytohormones and other bioactive metabolites [25].

**Figure 3:**
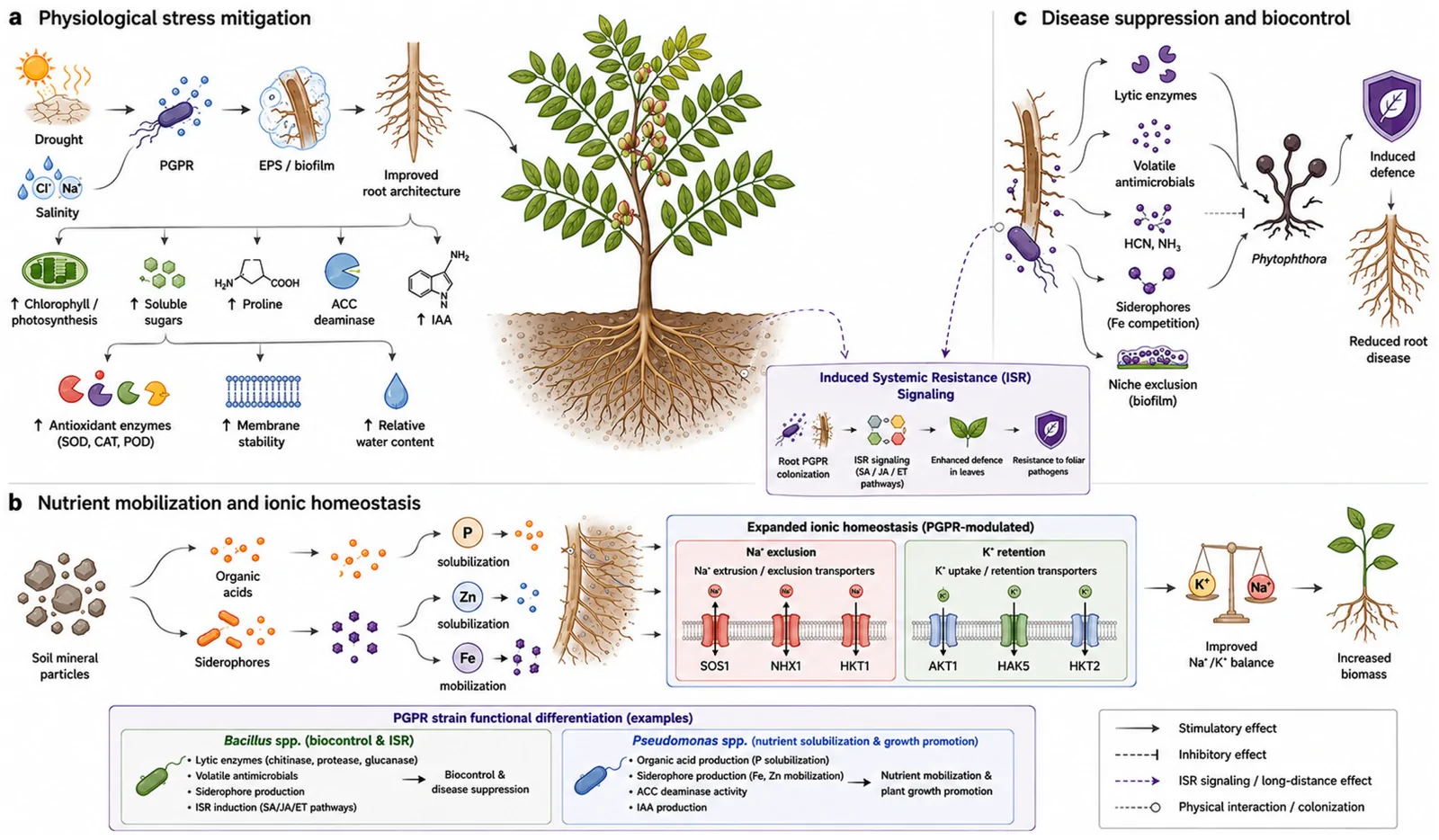
(**a**–**c**) Functional mechanisms through which plant growth-promoting rhizobacteria (PGPR) enhance plant performance under abiotic and biotic stress conditions in *P. vera*. The schematic summarizes the principal mechanisms by which PGPR improve plant adaptation to adverse environmental conditions. These include nutrient mobilization via phosphorus, potassium, zinc, and iron solubilization; siderophore production; biological nitrogen fixation; and phytohormone production, thereby enhancing nutrient acquisition and root development. The figure also illustrates PGPR-mediated biocontrol mechanisms, including competition for nutrients and ecological niches, production of antimicrobial compounds and lytic enzymes, and activation of induced systemic resistance (ISR), which collectively contribute to disease suppression. Under drought and salinity stress, PGPR promote osmotic adjustment, antioxidant defense, regulation of ethylene through 1-aminocyclopropane-1-carboxylate (ACC) deaminase activity, exopolysaccharide production, biofilm formation, and modulation of phytohormone signaling, thereby improving photosynthesis, water relations, nutrient uptake, and stress tolerance. Together, these mechanisms enhance plant growth, productivity, and resilience under adverse environmental conditions. ISR, induced systemic resistance; ACC, 1-aminocyclopropane-1-carboxylate; IAA, indole-3-acetic acid; EPS, exopolysaccharides; VOCs, volatile organic compounds; SOD, superoxide dismutase; POD, peroxidase; CAT, catalase; APX, ascorbate peroxidase; ROS, reactive oxygen species. https://chatgpt.com/s/m_6a3c071196108191b169b0e376613a4f, accessed on 3 August 2026 (Created with ChatGPT).

Nutrient mobilization is one of the main functional pathways through which PGPR influence plant nutritional status in the rhizosphere. This process is particularly relevant for nutrients with limited solubility or mobility in soil, such as P, K, Zn, and Fe, whose availability is strongly controlled by pH, mineral composition, and rhizosphere chemistry (Figure 3). Phosphate-solubilizing bacteria can release low-molecular-weight organic acids, including gluconic, citric, oxalic, malic, and succinic acids, which contribute to the dissolution of poorly soluble inorganic phosphates, such as calcium phosphates in neutral to alkaline soils and iron- or aluminum-bound phosphates in acidic soils [103,104,105]. Similar acidification and chelation processes have been described for K- and Zn-solubilizing bacteria, which can mobilize these nutrients from mineral sources or sparingly soluble compounds, particularly in alkaline and calcareous soils, where nutrient availability is constrained [106,107,108].

In addition to acid-mediated solubilization, siderophore production is a complementary mechanism for Fe acquisition and microbial competition in the rhizosphere (Figure 3). By chelating Fe, siderophore-producing bacteria may increase Fe availability under low solubility conditions and indirectly influence plant nutrition and microbial interactions [109]. However, the detection of these traits in vitro does not necessarily imply enhanced nutrient uptake under soil conditions. Their functional relevance depends on whether these metabolites are expressed in the rhizosphere, persist under prevailing edaphic conditions, and are translated into measurable changes in plant nutrient status. Therefore, nutrient-solubilizing and siderophore-producing PGPR should not be interpreted solely as biofertilizer candidates but also as microbial components capable of modifying nutrient gradients at the root–soil interface. This distinction is particularly important because laboratory-identified microbial traits do not always translate into equivalent responses in the field, where plant, soil, and environmental factors simultaneously influence microbial activity and nutrient dynamics.

In *P. vera*, the rhizosphere has been described as a reservoir of bacteria carrying traits associated with plant growth promotion, particularly under saline and water-deficit conditions. Several studies have reported stress-tolerant isolates capable of producing siderophores and solubilizing inorganic P and Zn sources, including Ca_3_(PO_4_)_2_ and ZnCO_3_ [109]. Similarly, *Pseudomonas fluorescens*, *Pantoea brenneri*, *Stenotrophomonas maltophilia*, and *Bacillus pumilus* have been identified as phosphate-solubilizing bacteria associated with pistachios [110,111]. Halotolerant and haloalkaliphilic bacteria with PGPR traits, including P and Zn solubilization, have been reported in pistachio-associated soils, suggesting their potential contribution to nutrient availability under saline–alkaline conditions [112]. Species belonging to Enterobacter and Enterococcus have also been identified among isolates with significant growth-promoting potential, primarily due to traits associated with nutrient mobilization under these conditions [113].

In addition to nutrient mobilization, bacterial inoculation has been associated with changes in mineral uptake in pistachio seedlings exposed to salinity. Although salinity reduced Zn concentration in shoots, PGPR inoculation increased Zn levels in roots at 2000 mg NaCl kg^−1^. In the same study, shoot P concentration also increased, suggesting that PGPR may partially mitigate the negative effects of salinity on nutrient acquisition [114]. The co-application of *Pseudomonas fluorescens* and vermicompost increased shoot dry weight and the uptake of P, K, Ca, and micronutrients compared with that in untreated plants [115]. Halotolerant PGPR have also been associated with changes in the ionic balance of pistachio seedlings under saline conditions. Specifically, *Alkalibacillus halotolerans*, *Virgibacillus marismortui*, and *Alkalibacillus haloalkaliphilus* reduced Na and Cl concentrations, as well as the Na^+^/K ratio, compared with uninoculated plants [116]. Similarly, *Pseudomonas* strains increased shoot Mn uptake under different salinity levels, whereas the combined application of PGPR and Mn increased shoot and root dry weights [117]. Together, these studies indicate that bacteria associated with the pistachio rhizosphere can influence nutrient acquisition and ionic homeostasis; however, the magnitude of these effects depends on the bacterial strain, stress intensity, plant developmental stage, and edaphic conditions (Table 1; Figure 2). Overall, the available evidence suggests that nutrient mobilization by PGPR is a potentially important mechanism contributing to pistachio adaptation in nutrient-limited and saline environments. However, further field-scale validation is required to assess the consistency of these responses under commercial production conditions.

### 4.2. Biocontrol Strategies

PGPR-mediated biocontrol involves direct and indirect mechanisms operating at the root–soil interface, including pathogen suppression, competition for nutrients and ecological niches, secretion of antimicrobial metabolites and lytic enzymes, and activation of host defense responses [118,119]. Functional traits commonly associated with this process include siderophore production, volatile organic compounds, hydrogen cyanide, lipopeptides, secondary metabolites, and antifungal activity [120,121]. These traits have been widely described in genera such as Bacillus and *Pseudomonas*, which include strains with antagonistic activity against fungal, bacterial, nematode, and viral pathogens in both annual and perennial crops [122]. However, the effectiveness of these mechanisms depends not only on the production of antimicrobial compounds but also on PGPR’s ability to establish, persist, and remain metabolically active in the rhizosphere under field conditions.

In Bacillus, biocontrol potential has been linked to the production of cyclic lipopeptides, including surfactin-related compounds, proteases, cellulases, and other antimicrobial substances that inhibit phytopathogenic fungi and bacteria [123,124]. In *Pseudomonas*, metabolites such as 2,4-diacetylphloroglucinol, pyoluteorin, and pyrrolnitrin have been associated with the suppression of soil-borne pathogens [125,126]. In addition, biosynthetic gene clusters encoding antimicrobial compounds, cyclic lipopeptides, hydrogen cyanide (HCN), and siderophores have been reported in bacterial biocontrol strains, further supporting their potential role in pathogen suppression [127].

Beyond direct antagonism, PGPR can contribute to plant protection by modulating host defense responses. Bacterial inoculation has been associated with increased phenylalanine ammonia-lyase activity, accumulation of phenolic compounds, lignin deposition, and enhanced activity of defense-related enzymes, including peroxidase and polyphenol oxidase [29,87]. In some cases, bacterial strains have been linked to the differential activation of salicylic acid- and jasmonic acid/ethylene-related signaling pathways [128]. These responses are consistent with the role of root-derived signaling molecules in recruiting and modulating beneficial bacteria in the rhizosphere [129]. Consequently, PGPR-mediated biocontrol should be viewed not only as direct pathogen suppression but also as a process involving microbiome-mediated enhancement of plant defense capacity and resilience to biotic stress (Figure 3). In addition to suppressing soil-borne pathogens, PGPR may protect aboveground tissues through induced systemic resistance (ISR). Following root colonization, beneficial bacteria can activate systemic defense responses mediated by jasmonic acid and ethylene signaling, resulting in enhanced expression of defense-related genes and increased accumulation of antimicrobial proteins and secondary metabolites in leaves and stems [29,128,129]. However, antimicrobial traits, biosynthetic genes, and defense-related markers should be interpreted as indicators of biocontrol potential rather than direct evidence of disease suppression unless validated through pathogen challenge assays under plant or soil conditions (Table 1). This distinction is particularly important because microbial traits identified under laboratory conditions do not always translate into equivalent disease suppression in greenhouse or orchard environments.

In *P. vera*, bacterial biocontrol has been investigated mainly against soil-borne pathogens affecting the root system (Table 2; Figure 3). PGPR strains isolated from the pistachio rhizosphere, such as *Bacillus velezensis*, have been reported to reduce infection caused by *Phytophthora drechsleri* by up to 87% compared to untreated controls. These effects are associated with lytic enzyme activity, particularly cellulases and proteases [130,131]. Similarly, *Bacillus subtilis* has shown antagonistic activity against *Phytophthora pistaciae*, with up to 70% inhibition of mycelial growth in vitro and an 80% reduction in seedling mortality under greenhouse conditions compared to controls inoculated only with the pathogen [132]. This activity concerns the production of volatile and non-volatile antimicrobial metabolites. Complementarily, formulations based on *Bacillus amyloliquefaciens* reduced root rot severity in pistachios by 70% [133]. Biocontrol-related traits have also been reported in other bacterial genera associated with the pistachio rhizosphere. Isolates of *Pseudomonas*, *Stenotrophomonas*, *Pantoea* and *Serratia* showed in vitro antagonistic activity against bacterial phytopathogens, including *Pseudomonas syringae* pv. syringae and *Pseudomonas tolaasii* [111]. In susceptible pistachio plants challenged with *Phytophthora parsiana*, inoculation with *Pseudomonas fluorescens* was associated with the upregulation of genes involved in defense regulation [134]. These findings suggest that PGPR may contribute to disease suppression not only through direct antagonism but also by activating and priming the plant defense pathways.

Multiple biological and environmental factors also influence reported differences in the biocontrol performance of PGPR. Although several strains consistently suppress *Phytophthora* spp. and other pathogens, the magnitude of disease reduction varies depending on rhizosphere colonization efficiency, persistence under saline or drought conditions, production of antimicrobial metabolites, and competition with the native microbiota. In addition, pathogen pressure, soil properties, inoculum formulation, and environmental stress can modify the expression of bacterial biocontrol traits. Consequently, inconsistent results among studies most likely reflect differences in experimental context rather than an absence of biocontrol potential.

However, further studies are required to evaluate the consistency and efficacy of these biocontrol mechanisms under commercial conditions. *Pseudomonas chlororaphis* has been shown to suppress *Phytophthora drechsleri* in planta, reducing disease severity by up to 87%. This activity has been linked to biocontrol-related traits, such as HCN and siderophore production, which may contribute to pathogen inhibition through metabolic interference and competition for Fe, respectively [131]. A recent study also characterized epiphytic and endophytic bacteria, *Pseudomonas chlororaphis* and *Bacillus mojavensis*, isolated from pistachio-associated tissues. These isolates differed significantly in the production of biocontrol-related metabolites, including siderophores, proteases, HCN, and ammonia, and exhibited antagonistic activity against the phytopathogenic fungi *Botryosphaeria dothidea*, *Neofusicoccum parvum*, and *Neoscytalidium dimidiatum* [135]. Taken together, the available evidence indicates that PGPR associated with the pistachio rhizosphere can suppress pathogens through a combination of antimicrobial activity, resource competition, and induction of host defense responses (Table 2; Figure 3). Nevertheless, additional field-based studies are required to determine the consistency and long-term effectiveness.

Although evidence specifically addressing foliar fungal pathogens and vascular bacterial diseases in pistachio remains scarce, these mechanisms may help limit the development of diseases caused by pathogens such as *Septoria* spp., *Botryosphaeria* spp., and *Xylella fastidiosa* [28]. Further research is needed to determine the relevance of these systemic defense mechanisms under commercial orchard conditions.

### 4.3. Mitigation of Water Stress and Salinity

Plant responses to drought are determined not only by intrinsic physiological adjustments but also by interactions between the roots and rhizosphere microorganisms. PGPR can contribute to drought tolerance by modulating root growth, osmotic adjustment, nutrient uptake, antioxidant activity, and stress-related signaling pathways [136]. Consequently, PGPR are increasingly recognized as important biological mediators of plant adaptation to water-limited environments, where multiple physiological and biochemical processes must be coordinated to maintain growth and productivity (Table 2; Figure 3).

Among these mechanisms, ACC deaminase activity is particularly relevant, as it reduces stress-induced ethylene accumulation by cleaving ACC, the immediate precursor of ethylene, into ammonia and α-ketobutyrate [137]. Under drought stress, ACC deaminase-producing PGPR may reduce ethylene accumulation and modulate plant hormonal balance, including abscisic acid (ABA)- and auxin-related responses. These changes have been associated with increased root biomass, chlorophyll color index, net photosynthetic rate, stomatal conductance, and relative water content, as well as enhanced activity of antioxidant enzymes such as catalase (CAT), ascorbate peroxidase (APX) and superoxide dismutase (SOD) [138,139].

By reducing stress-induced ethylene accumulation, ACC deaminase-producing PGPR can indirectly modulate drought response processes, including aquaporin activity and the expression of stress-protective proteins. These effects may support water uptake, hydraulic conductance, and water-use efficiency under water deficit conditions [140]. In addition, PGPR inoculation was associated with increased nutrient accumulation, relative water content, photosynthetic pigment content, and leaf protein and soluble sugar concentrations under drought stress (Figure 3). ACC deaminase activity has also been linked to higher chlorophyll a and b contents, photosynthetic rate, fruit weight, and N, P, and K concentrations in fruits under drought conditions [16].

PGPR-treated plants may also accumulate higher levels of osmoprotective compounds, such as soluble sugars, soluble proteins, and proline [141,142], while further modulating hormonal signaling and water transport under water-deficit conditions [143] (Figure 3).

Integrating metabolomic and transcriptomic approaches has provided new insights into how plants and their associated microbiomes respond to abiotic stress. Under water deficit, root exudates mediate the exchange of metabolites, osmolytes, antioxidant enzymes, and phytohormones involved in the activation of defense responses. Jasmonic acid (JA) and salicylic acid (SA) are key components of the signaling networks that regulate PGPR–plant interactions [144]. These pathways contribute to rhizosphere colonization, microbial community assembly, and the activation of induced systemic resistance. However, the relative involvement of JA- and SA-dependent responses varies according to the microbial strain, host genotype, and environmental conditions. Also, the expression of ion transporters such as HKT, NHX, and SOS1, together with antioxidant genes including SOD, CAT, APX, and GR, is modulated to support ionic homeostasis and mitigate oxidative stress [145,146]. Advanced metabolomic approaches have been used to investigate the relationships between specific metabolites in drought-stressed (including acetyl-galactoside, luteolin 7-O-(2-apiosyl-6-malonyl)-glucoside, benzoic acid, malate, 2,4-dihydroxybenzoic acid, and hesperidin) and the abundance of particular bacterial groups associated with the plant microbiome [147]. Emerging technologies, including synthetic biology, hold promise for tailoring PGPR strains and optimizing their performance, while their integration into precision agriculture systems may enable more targeted and efficient stress management [148].

Exopolysaccharide production and biofilm formation are particularly relevant because they can enhance bacterial adhesion to root surfaces, improve rhizosphere colonization, and promote the establishment of microbial communities that persist under low soil water availability [136]. In addition, PGPR-mediated hormonal modulation may contribute to drought adaptation by producing indole-3-acetic acid (IAA). Bacterial IAA can stimulate root system development by promoting lateral root formation and root hair proliferation, thereby increasing the soil volume the plant explores. This response may improve water and nutrient acquisition under water-limited conditions [149]. Furthermore, enhanced root system development may increase the plant’s capacity to exploit heterogeneous soil water and nutrient patches, thereby improving resilience under prolonged drought [149,150].

VOCs represent another mechanism through which bacteria trigger systemic responses in plants under water-deficit conditions [151]. These compounds complement the physiological and molecular mechanisms described above by modulating phytohormonal signaling and contributing to the maintenance of photosynthetic performance and cellular homeostasis during stress. Among the VOCs most frequently associated with plant stress responses are dimethyl disulfide, 2,3-butanediol, and 2-pentylfuran [152,153].

In pistachios, inoculation of seedlings with stress-tolerant PGPR isolates, including *Staphylococcus sciuri*, *Zobellella denitrificans*, and *Arthrobacter endophyticus*, has been reported to increase plant growth and improve photosynthetic parameters under saline and drought stress, either individually or in combination [154]. Under saline stress, native pistachio-associated PGPR can enhance plant performance by attenuating stress-related physiological and biochemical alterations. Reported responses include reduced phenol accumulation, increased chlorophyll content, and lower carotenoid levels. Furthermore, IAA-producing bacteria may promote root growth and lateral root formation, thereby increasing soil exploration and improving water and nutrient uptake under saline conditions [113].

Genotype-specific pathways shape ionic homeostasis under saline stress and can be modulated by PGPR inoculation. The maintenance of high chlorophyll levels in some rootstocks under moderate salinity suggests that certain genotypes may preserve photosynthetic pigment stability despite ionic stress (Table 2). The formulation of PGPR under moderate salinity improved ionic balance, stimulation of root growth, leaf physiology, and nutrient uptake in pistachio rootstocks [7].

Inoculation with *Paenarthrobacter nitroguajacolicus* improved pistachio seedling performance under salinity, resulting in higher total chlorophyll content and greater accumulation of soluble sugars and proline than in uninoculated controls [112]. These responses are consistent with the functional traits described for this isolate, including ACC deaminase activity, IAA production, and solubilization of P and Zn.

Similarly, PGPR application under salinity improved membrane stability, osmotic adjustment, and oxidative balance, as reflected by increased RWC, MSI, and sucrose levels, along with reduced MDA accumulation in pistachio seedlings [110]. These responses are consistent with the physiological and molecular mechanisms discussed above and further support the role of PGPR in enhancing plant resilience under abiotic stress conditions (Table 2; Figure 3).

**Table 2:** Functional traits and plant responses associated with PGPR inoculation in *P. vera* under abiotic and biotic stress conditions.

Functional Component	PGPR Strain	Pistachio Cultivar	Stress Condition	Inoculant Dosage	Specific PGPR-Mediated Response or Trait	Ref.
Drought/salinity tolerance and physiological stress mitigation	*Staphylococcus sciuri*, *Zobellella denitrificans*, and *Arthrobacter endophyticus*	*P. vera* cv. Badami-Riz-e-Zarand seedlings	Combined salinity (0, 1000 and 2000 mg NaCl kg^−1^ soil) and drought (2-, 4-, and 6-day irrigation intervals)	1 mL bacterial suspension (10^8^ CFU mL^−1^) per seed	Improved shoot and root growth, relative water content (RWC), water-use efficiency (WUE), chlorophyll and carotenoid contents, chlorophyll fluorescence (Fv/Fm), gas exchange (Pn, Gs, E, and Ci), and K^+^/Na^+^ homeostasis, thereby enhancing tolerance to combined salinity and drought stress.	[154]
	*Paenarthrobacter nitroguajacolicus P1*	*P. vera* cv. Akbari seedlings	Moderately saline soil conditions	1 mL bacterial suspension (10^8^ CFU mL^−1^) per seed	Enhanced stress tolerance by increasing total chlorophyll, soluble sugars, proline, anthocyanins, and phenolic compounds, while promoting physiological adjustment through ACC deaminase activity, IAA production, and P/Zn solubilization	[112]
	Native halotolerant PGPR isolates, including *Enterobacter hormaechei* subsp. hoffmannii DU22 and *Enterococcus durans* DU4.	*P. vera* seedlings	Moderately saline–alkaline soil conditions (EC 6.8 dS m^−1^)	Seed immersion for 2 h in bacterial suspension (10^8^ CFU mL^−1^)	Improved physiological performance by increasing chlorophyll, carotenoids, phenolic compounds, and anthocyanins, reflecting enhanced tolerance to saline conditions through multiple plant growth-promoting traits, including IAA production, halotolerance, exopolysaccharide production, and nutrient solubilization.	[113]
	PGPR isolates associated with the pistachio rhizosphere	Seven pistachio rootstocks (*P. khinjuk* A and B, *P. atlantica*, *P. mutica*, *P. terebinthus*, and *P. vera* cvs. Siirt and Kırmızı)	Salinity (0, 100 and 200 mM NaCl)	Commercial formulation (1 × 10^6^ CFU mL^−1^), applied at 15, 30, and 45 days after salinity treatment initiation	Partially alleviated moderate salinity stress by improving shoot and root growth, relative water content, stomatal conductance, chlorophyll index, K^+^/Na^+^ homeostasis, and reducing Na^+^ accumulation in responsive rootstocks.	[110]
	PGPR inoculation in pistachio rootstocks	*P. vera* cv. Badami seedlings	Salinity (0, 1000 and 2000 mg NaCl kg^−1^ soil), with or without Zn (5 mg kg^−1^ soil)	1 mL bacterial suspension (10^8^ CFU mL^−1^) per seed	Enhanced tolerance to salinity by improving shoot and root growth, leaf area, relative water content, membrane stability, nutrient uptake, K^+^/Na^+^ balance, and reducing oxidative damage (lower MDA), particularly when combined with Zn application.	[7]
Nutrient solubilization, mobilization and ionic homeostasis	*Staphylococcus sciuri, Zobellella denitrificans,* and *Arthrobacter endophyticus*	*P. vera* cv. Badami-Riz-e-Zarand seedlings	Combined salinity (0, 1000 and 2000 mg NaCl kg^−1^ soil) and drought (2-, 4-, and 6-day irrigation intervals)	1 mL bacterial suspension (10^8^ CFU mL^−1^) per seed	Enhanced P and Zn solubilization, siderophore production, and K^+^ acquisition while reducing Na^+^ accumulation, thereby improving nutrient uptake and ionic homeostasis under combined salinity and drought stress.	[154]
	Endophytic bacterial isolates belonging to *Pseudomonas, Stenotrophomonas, Bacillus, Pantoea,* and *Serratia*	Wild pistachio (*P. atlantica* subsp. kurdica)	Non-stress conditions	Non-reported	Exhibited multiple nutrient-mobilizing traits, including phosphate solubilization, siderophore production, atmospheric nitrogen fixation, and phytohormone (IAA and gibberellin) production, highlighting their potential to improve nutrient availability and acquisition in pistachio.	[111]
	Halotolerant *Paenarthrobacter nitroguajacolicus P1*	*P. vera* seedlings	Saline–alkaline conditions	1 mL bacterial suspension (10^8^ CFU mL^−1^) per seedling	Exhibited strong phosphate and zinc solubilization, IAA production, and hydrolytic enzyme activities, enhancing nutrient bioavailability and mineral acquisition for pistachio seedlings.	[112]
	Native halotolerant PGPR, including *Enterobacter hormaechei subsp. hoffmannii DU22* and *Enterococcus durans DU4*.	*P. vera* seedlings	Slightly saline–alkaline soil	Seed inoculation with selected native strains (10^8^ CFU mL^−1^)	Improved nutrient mobilization through phosphate solubilization, IAA production, exopolysaccharide synthesis, and ammonia production, while enhancing nutrient availability and adaptation to saline–alkaline soils.	[113]
	Fluorescent *Pseudomonas* isolates (pf1, pf2 and pf3)	*P. vera* cv. Badami seedlings	Salinity (0, 1000 and 2000 mg NaCl kg^−1^ soil) with or without Zn supplementation	1 mL bacterial suspension (10^8^ CFU mL^−1^) per seed.	Enhanced uptake of P, K, Ca, Mg, and Zn, increased K^+^/Na^+^ and Ca^2+^/Na^+^ ratios, and reduced Na^+^ and Cl^−^ accumulation, thereby improving nutrient acquisition and ionic homeostasis under salinity stress.	[110]
	Fluorescent *Pseudomonas* isolates D12 and D16.	*P. vera* cv. Qazvini seedlings	Non-stress conditions	Bacterial inoculation combined with 0, 2, or 4% (w/w) vermicompost	Promoted nutrient mobilization by increasing shoot uptake of P, K, Ca, Fe, Zn, Mn, and Cu, with synergistic improvements in mineral acquisition when combined with vermicompost.	[115]
	*Virgibacillus marismortui, Alkalibacillus haloalkaliphilus,* and their mixed inocula.	*P. vera* cvs. Akbari and Daneshmandi	Saline–alkaline orchard soils (8, 12 and 16 dS m^−1^)	Non-reported	Enhanced nutrient acquisition and ionic homeostasis by increasing P, K and B concentrations, reducing Na^+^ and Cl^−^ accumulation, improving the K^+^/Na^+^ ratio, and promoting ammonia production, ACC deaminase activity, and IAA synthesis under saline–alkaline conditions.	[116]
	Fluorescent *Pseudomonas isolates (pf0, pf1, pf2 and pf3)*	*P. vera* L. seedlings	Salinity stress (0, 1000 and 2000 mg NaCl kg^−1^ soil) with Zn supplementation (0 or 5 mg Zn kg^−1^ soil)	1 mL bacterial suspension (10^8^ CFU mL^−1^) per seed	Enhanced nutrient mobilization by increasing P, K, Ca, Mg and Zn uptake, improving K^+^/Na^+^ and Ca^2+^/Na^+^ ratios, and reducing Na^+^ and Cl^−^ accumulation, thereby promoting nutrient acquisition and ionic homeostasis under salinity stress.	[117]
Disease suppression and biocontrol	*Bacillus velezensis* (encapsulated in sodium alginate–gelatin nanocomposite enriched with carbon nanotubes and SiO_2_ nanoparticles)	*P. vera* seedlings	*Phytophthora drechsleri* (pistachio gummosis)	Encapsulated formulation (≈10^7^ CFU mL^−1^ after encapsulation)	Infection was reduced by up to 87% compared to untreated controls. The effect was associated with lytic enzyme activity, particularly cellulases and proteases.	[130]
	*Native Bacillus subtilis strains BSIPR7, BSIPR22* and *BSIPR35*	*P. vera* cv. Sarakhs seedlings	*Phytophthora pistaciae* (pistachio gummosis)	200 mL bacterial suspension (2 × 10^8^ CFU g^−1^)	Suppressed Phytophthora crown and root rot through strong antagonistic activity mediated by diffusible and volatile antimicrobial metabolites, significantly reducing pathogen growth and seedling mortality, while demonstrating tolerance to drought, salinity and high temperature, supporting their suitability as biological control agents under orchard conditions.	[131]
	*Pseudomonas chlororaphis VUPF5* (encapsulated in sodium alginate–soy protein isolate microcapsules)	*P. vera* cv. Badami-Riz-e-Zarand seedlings	*Phytophthora drechsleri* (pistachio gummosis)	4 g encapsulated inoculum pot^−1^	Enhanced suppression of pistachio gummosis through sustained release of viable bacterial cells producing siderophores, HCN, cellulase, protease, phosphate-solubilizing activity, volatile antifungal compounds, and IAA, resulting in long-lasting pathogen inhibition and improved disease control.	[132]
	Endophytic bacterial isolates belonging to *Pseudomonas protegens, Stenotrophomonas maltophilia, Bacillus pumilus, Serratia plymuthica,* and *Pantoea brenneri.*	Wild pistachio (*P. atlantica* subsp. kurdica)	In vitro antagonism against *Pseudomonas syringae* pv. syringae and *Pseudomonas tolaasii*	Non-reported	Exhibited biocontrol potential through the production of siderophores, proteases, HCN, and phytohormones, together with strong antagonistic activity against bacterial phytopathogens, highlighting their potential as biological control agents for pistachio.	[111]
	*Pseudomonas fluorescens* VUPF5	*P. vera* cv. Sarakhs (susceptible) and Badami-Rize Zarandi (resistant) seedlings	Gummosis caused by *Phytophthora parsiana*	40 mL bacterial suspension (10^8^ CFU g^−1^ soil	Inoculation was associated with the upregulation of genes involved in defense regulation, suggesting activation of plant defense responses.	[134]
	*Pseudomonas chlororaphis VUPF5* (encapsulated in sodium alginate–soy protein isolate microcapsules)	*P. vera* cv. Badami-Rize-Zarand seedlings	Gummosis caused by *Phytophthora drechsleri*	40 mL bacterial suspension (10^10^ CFU mL^−1^) or 4 g microcapsules per pot	Displayed multiple PGPR traits, including phosphate solubilization, siderophore, IAA, HCN, cellulase and protease production, together with strong antifungal activity. Microencapsulation improved bacterial survival and controlled release, resulting in 87% disease suppression in greenhouse-grown pistachio seedlings, outperforming the free bacterial inoculum.	[131]
	*Pseudomonas chlororaphis NKA10ep, Bacillus mojavensis NKA7en, and Bacillus pumilus NKC3en*	Healthy *P. vera* tissues (endophytic and epiphytic isolates)	Dieback pathogens (*Botryosphaeria dothidea*, *Neofusicoccum parvum*, and *Neoscytalidium dimidiatum*)	Not applicable (in vitro screening)	Isolates produced biocontrol-related metabolites, including siderophores, proteases, HCN and ammonia, and showed antagonistic activity against fungal pathogens.	[135]

## 5. Endophytic Microorganisms Associated with Pistacia

Endophytic microorganisms colonize internal plant tissues without causing visible disease symptoms and comprise taxonomically and functionally diverse bacterial and fungal communities. However, current knowledge of endophytic communities in Pistacia is based predominantly on culture-dependent isolation and in vitro functional characterization.

One of the available studies isolated 61 culturable bacterial endophytes from surface-disinfected leaves and stems of healthy wild *Pistacia atlantica* trees collected in Iran. The isolates were assigned to five genera: *Pantoea*, *Bacillus*, *Pseudomonas*, *Serratia*, and *Stenotrophomonas*. Several isolates exhibited traits associated with plant growth promotion and antagonistic activity, including indole-3-acetic acid production, phosphate solubilization, siderophore production, and inhibition of *Pseudomonas syringae* and *Pseudomonas tolaasii* [111]. These findings demonstrate the functional potential of the recovered isolates under in vitro conditions. However, the study did not establish their persistence within Pistacia tissues or validate their beneficial effects under greenhouse or orchard conditions.

Fungal endophytes have also been investigated in *P. vera*. In one study, fungal isolates recovered from pistachio tissues were screened for functional traits, including antifungal activity and phosphate solubilization [155]. These results indicate that the endophytic mycobiota of pistachio may contain strains with potential relevance for nutrient mobilization and biological control. Nevertheless, as with the bacterial endophytes, the available evidence is based mainly on laboratory screening and does not yet demonstrate consistent beneficial effects in inoculated pistachio plants.

A major limitation of culture-dependent approaches is that they recover only a fraction of the endophytic community and may exclude microorganisms that are difficult or impossible to cultivate under standard laboratory conditions. Moreover, functional traits detected in vitro may not be expressed within plant tissues or under soil and field conditions. Therefore, results derived exclusively from laboratory screening should be interpreted cautiously and clearly distinguished from responses validated through controlled inoculation experiments. Future research should integrate culture-dependent isolation with amplicon sequencing, metagenomics, colonization tracking, and in planta validation. Such an approach would enable a more comprehensive characterization of bacterial and fungal endophytes associated with different Pistacia genotypes and tissues. It would also help determine whether candidate strains can colonize and persist within the host and whether they consistently improve plant growth, nutrient acquisition, stress tolerance, or disease suppression under greenhouse and field conditions.

## 6. AMF–PGPR Interactions: Toward Functional Synergy

Co-inoculation with AMF and PGPR is a promising biotechnological strategy for enhancing plant growth, mineral nutrition, and stress tolerance [156]. However, its effectiveness depends on the compatibility of specific microorganisms, host plants, and edaphic conditions; therefore, the observed response should not be automatically interpreted as functional synergy. In this context, combinations of positively interacting PGPR and AMF have been proposed as efficient and cost-effective alternatives for improving plant tolerance to stress. However, their effects may vary among crops, genotypes, soils, and microbial formulations [157]. Consequently, understanding the mechanisms governing AMF–PGPR compatibility is essential for predicting when co-inoculation will result in additive, complementary, or truly synergistic effects.

Although co-inoculation frequently produces additive or synergistic responses, neutral or even antagonistic interactions have also been reported. Differences in microbial compatibility, competition for plant-derived carbon resources, temporal patterns of root colonization, recruitment of bacteria within the mycorrhizosphere, and the functional complementarity of the inoculated microorganisms likely explain these contrasting outcomes. Moreover, soil physicochemical properties, nutrient availability, stress severity, inoculation timing, inoculum composition, and plant developmental stage strongly influence the establishment and effectiveness of microbial consortia. Therefore, the success of co-inoculation depends not only on combining beneficial microorganisms but also on selecting compatible partners that can function under specific environmental conditions.

From a metabolic perspective, AMF–PGPR co-inoculation can induce biochemical reprogramming in the roots and rhizosphere. In specific microorganism–host plant combinations, pathways associated with flavonoid and other phenolic compound biosynthesis have been upregulated, which may promote plant–microorganism signaling, root colonization, and the activation of adaptive responses [158]. In addition, AMF–PGPR co-inoculation has been associated with changes in root secondary metabolite accumulation, including variations in verbascoside concentration [159]. Likewise, AMF–PGPR combinations have been reported to reduce electrolyte leakage and lipid peroxidation, while increasing the accumulation of phenols, flavonoids, ABA, and IAA, suggesting integrated regulation among secondary metabolism, hormonal signaling, and antioxidant protection [26].

The interaction between AMF and PGPR can lead to morphological, physiological, and nutritional improvements in plant growth. Co-inoculation has been reported to enhance morphological traits and promote greater uptake of macronutrients, such as N, P, and K, possibly due to the complementarity between bacterial nutrient mobilization in the rhizosphere and AMF-mediated hyphal absorption [158]. In seedlings, this interaction also increases root colonization intensity, vegetation index, total chlorophyll content, and leaf area [160]. Moreover, the co-application of PGPR and AMF significantly increased root biomass and the abundance of potentially beneficial bacterial genera in roots compared with uninoculated treatments and individual inoculations [161]. These findings support the idea that AMF and PGPR occupy complementary functional niches, allowing simultaneous improvements in nutrient acquisition, root development, and plant physiological performance.

The improvement in plant nutrition under co-inoculation can be explained by mechanisms that operate in an integrated manner within the host plant’s rhizosphere. PGPR may contribute to the solubilization of phosphate, zinc, and other poorly available nutrients by producing organic acids, siderophores, and altering local pH. In contrast, AMF expand the volume of soil explored through extraradical hyphae and facilitate the transport of phosphorus, nitrogen, and other micronutrients to the plant. In this regard, the benefits attributed to AMF and PGPR have been linked to increased soil nutrient availability, suggesting that part of the plant response may be mediated by changes in the chemical and biological fertility of the rhizosphere [162]. Furthermore, the combination of AMF and PGPR may increase glomalin content, improve root architecture, stimulate plant growth, and enhance phosphorus nutrition [163].

Co-inoculation may also modify soil biochemical processes involved in nutrient cycling. The joint application of PGPR and AMF has increased enzymatic activities, including urease, alkaline phosphatase, β-glucosidase, and dehydrogenase, indicating a potential improvement in soil functional quality and nutrient mineralization [164]. Similarly, AMF + PGPR treatments have increased phosphatase, dehydrogenase, and peroxidase activities, with reported increases of 117–221%, 89–147%, and 71–85%, respectively, depending on the evaluated condition [165]. These responses suggest that co-inoculation may affect not only the plant but also soil microbial and enzymatic activity; however, its agronomic impact will depend on inoculant persistence and the native microbiota. This is particularly relevant because improvements in soil function may yield longer-lasting benefits than those derived solely from the short-term stimulation of plant growth.

Under drought stress, PGPR may contribute to plant tolerance through EPS production, biofilm formation, ACC deaminase activity, ethylene regulation, phytohormone production, osmoprotectant accumulation, and root architecture modification [138]. In parallel, AMF may enhance the exploration of soil micropores through extraradical hyphae, improve plant water potential, increase water-use efficiency, and sustain stomatal conductance, RWC, and photosynthetic protection. In this context, the combination of mycorrhizae and PGPR has been associated with the regulation of physiological and biochemical processes that enable plants to cope with drought, reinforcing the importance of analyzing these interactions within the soil–plant–microbiome continuum [166]. Similarly, co-inoculation has been reported to mitigate the negative effects of drought on biomass, mesophyll conductance, and RWC [167].

Responses to drought also involve osmotic, photosynthetic, and antioxidant adjustments. Inoculation with PGPR, AMF, and concomitant microorganisms improved photosynthetic pigments, proline content, and RWC, thereby promoting the restoration of growth and biomass under water stress [168]. In addition, PGPR–AMF co-inoculation may promote faster recovery after rehydration, which is associated with greater growth, improved stomatal conductance, and enhanced nutrient uptake [169]. These observations suggest that co-inoculation may influence not only resistance to drought but also resilience, defined as the capacity of plants to recover following stress relief.

Under saline conditions, AMF–PGPR interactions may contribute to ionic homeostasis and redox balance. In rapeseed, the joint application of PGPR and AMF has been associated with greater salinity tolerance, attributed to improvements in enzymatic antioxidant activity, nutritional status, and regulation of ionic balance [170]. Specifically, inoculation with *Rhizophagus intraradices* and/or halotolerant PGPR, such as *Bacillus subtilis*, has been linked to improved plant growth, enhanced functional biochemical synthesis, and endogenous ABA signaling during short- and long-term responses to salt stress [169]. Moreover, co-inoculation with AMF and *Pseudomonas* sp. under salinity reduced sodium accumulation in shoots, electrolyte leakage, and MDA levels [171].

The mitigation of salt stress by AMF and PGPR has also been associated with greater potassium retention and lower sodium and chloride accumulation, thereby reducing ionic imbalance. These responses, together with increased chlorophyll content and improved plant water relations, highlight the potential of these biostimulants to sustain photosynthesis and plant resilience under saline conditions [172]. Similarly, inoculation has been shown to improve the activity of antioxidant enzymes, such as superoxide dismutase, catalase, and peroxidase, while reducing oxidative damage by decreasing lipid peroxidation and hydrogen peroxide levels [173].

Both direct and indirect mechanisms can explain the disease-suppressing effects of AMF and PGPR. In addition to competing for niches and nutrients and inducing ISR, PGPR may produce siderophores, antibiotics, lytic enzymes, and other antimicrobial compounds [25]. In turn, AMF may modify root exudates, modulate the structure of the rhizosphere microbial community, improve plant nutritional status, and indirectly reduce susceptibility to pathogens. The multifunctionality of these microorganisms has been associated with an 85–90% reduction in disease severity, attributed to interactions between mycorrhizae and rhizobacteria [174].

AMF–PGPR interactions may also modify the composition of root-associated microbiomes. AMF have been observed to display positive associations with beneficial bacterial taxa, including *Rhizobium*, *Pseudomonas*, and *Streptomyces*, suggesting that mycorrhizal symbiosis may favor the assembly of functionally relevant bacterial communities in the rhizosphere and hyphosphere [175]. This emerging evidence suggests that the benefits of co-inoculation may extend beyond the introduced microorganisms, potentially influencing the structure and function of the broader root-associated microbiome.

Although studies in other crop species largely support the mechanisms and benefits described above, direct evidence of AMF–PGPR co-inoculation in *P. vera* remains limited. The available studies on *P. vera* cv. Qazvini seedlings were mainly evaluated for the combination of *Glomus mosseae* (currently referred to in many taxonomic contexts as *Funneliformis mosseae*) and *Pseudomonas fluorescens* under different irrigation regimes, reporting improvements in vegetative growth, leaf area, and parameters associated with the photochemical yield of PSII [176]. Although these results are promising, the scarcity of studies on pistachios highlights an important knowledge gap in the literature. Future research should determine whether the synergistic effects reported in other crops can be consistently reproduced across pistachio cultivars, environmental conditions, rootstocks, and orchard management systems.

Advances in high-throughput sequencing technologies are also improving our understanding of how microbial inoculants influence native rhizosphere and mycorrhizosphere communities. Amplicon sequencing and other microbiome-based approaches have demonstrated that AMF and PGPR inoculation can alter microbial community composition, modify microbial interaction networks, and influence the abundance of functional microbial groups involved in nutrient cycling and plant health. Although such studies remain scarce in pistachio, integrating microbiome profiling with transcriptomic, proteomic, and metabolomic approaches represents a promising direction for identifying the mechanisms governing microbial establishment, persistence, and functional performance under field conditions.

## 7. Challenges for Orchard-Scale Implementation and Future Perspectives

Despite the growing body of evidence supporting the beneficial effects of arbuscular mycorrhizal fungi (AMF) and plant growth-promoting rhizobacteria (PGPR) in pistachio, several challenges continue to limit their successful implementation in commercial orchards. One of the main constraints is the establishment and persistence of introduced microorganisms within native soil microbial communities. In many pistachio-growing regions, alkaline and calcareous soils harbor well-adapted indigenous microbial populations that may compete with introduced inoculants for ecological niches and root colonization, thereby reducing their long-term effectiveness.

Formulation characteristics, carrier materials, application methods, and inoculation timing also influence the agronomic performance of microbial inoculants. Strategies such as nursery inoculation, root dipping before transplanting, soil drenching, and granular formulations may differ substantially in their capacity to establish stable microbial populations under field conditions. However, comparative studies evaluating these approaches in commercial pistachio orchards remain limited.

Another important challenge is the long-term persistence and functional stability of inoculated microorganisms. Because pistachio is a perennial crop with a productive lifespan spanning several decades, microbial inoculants should remain active across multiple growing seasons to provide sustained agronomic benefits. Long-term field studies are therefore required to determine the persistence, ecological stability, and functional performance of AMF and PGPR under diverse environmental and management conditions.

Alternate bearing represents another major challenge for commercial pistachio production. Although improved nutrient acquisition, enhanced photosynthetic performance, and greater tolerance to abiotic stress could contribute to a more balanced allocation of carbon and mineral nutrients between vegetative growth and reproductive development, direct experimental evidence demonstrating that AMF or PGPR reduce biennial yield fluctuations in pistachio remains scarce. Addressing this knowledge gap should be considered a priority for future research.

Overall, future investigations should prioritize long-term, multi-site field experiments integrating microbial ecology, soil properties, rootstock genotype, orchard management practices, and agronomic performance. Such studies will be essential for developing robust microbiome-based strategies that can be reliably implemented under commercial pistachio production systems.

## 8. Concluding Remarks

Arbuscular mycorrhizal fungi and plant growth-promoting rhizobacteria play important and complementary roles in enhancing the performance of *P. vera* under both abiotic and biotic stress conditions. The available evidence indicates that these microorganisms contribute to plant adaptation through multiple mechanisms. While AMF primarily improve phosphorus acquisition, water uptake, and soil structural stability, PGPR contribute to nutrient mobilization, biological control, phytohormone-mediated responses, and stress alleviation through various rhizosphere processes.

The growing body of research reviewed here supports the notion that the rhizosphere microbiome is an important component of pistachio resilience under drought, salinity, nutrient limitation, and disease pressure. Emerging evidence suggests that AMF–PGPR co-inoculation may provide complementary benefits by simultaneously improving plant nutrition, stress tolerance, soil functionality, and microbial interactions within the root environment. However, the magnitude and consistency of these responses remain highly dependent on the microbial strain identity, host genotype, environmental conditions, soil characteristics, and management practices.

Despite the promising results reported to date, most studies have been conducted under controlled greenhouse or nursery conditions, often using a limited number of cultivars, rootstocks, and microbial inoculants. Consequently, the translation of these findings into commercial orchards remains insufficiently validated in the literature. Future research should prioritize long-term field trials, multi-location evaluations, characterization of native pistachio-associated microbiomes, and a deeper understanding of AMF–PGPR interactions within the soil–plant–microbiome continuum. Integrating microbial ecology, functional traits, and agronomic performance will be essential to identify robust inoculant combinations and develop reliable microbiome-based strategies. Addressing these knowledge gaps will improve the consistency, scalability, and field validation of microbial technologies, thereby supporting more resilient and sustainable pistachio production under increasingly challenging environmental conditions.

## Data Availability

No new data were created or analyzed in this study. Data sharing is not applicable to this article.

## Abbreviations

The following abbreviations are used in this manuscript:

ABA	Abscisic acid
ACC	1-Aminocyclopropane-1-carboxylic acid
ACP	Acid phosphatase
ALP	Alkaline phosphatase
AMF	Arbuscular mycorrhizal fungi
APX	Ascorbate peroxidase
CAT	Catalase
EPS	Exopolysaccharides
Fv/Fm	Maximum quantum yield of PSII photochemistry
Fv′/Fm′	Efficiency of excitation capture by open PSII reaction centers
GR	Glutathione reductase
GSH	Reduced glutathione
GSSG	Oxidized glutathione
HCN	Hydrogen cyanide
IAA	Indole-3-acetic acid
ISR	Induced systemic resistance
JA	Jasmonic acid
LAP	Leucine aminopeptidase
MAPK	Mitogen-activated protein kinase
MDA	Malondialdehyde
MSI	Membrane stability index
NAG	β-N-acetylglucosaminidase
NH_4_^+^	Ammonium
NHX	Na^+^/H^+^ exchanger
NO_3_^−^	Nitrate
NPQ	Non-photochemical quenching
PAL	Phenylalanine ammonia-lyase
PGPR	Plant growth-promoting rhizobacteria
Pn	Net photosynthetic rate
POD	Peroxidase
PPO	Polyphenol oxidase
PSII	Photosystem II
ROS	Reactive oxygen species
RWC	Relative water content
SA	Salicylic acid
SOD	Superoxide dismutase
SOS1	Salt overly sensitive 1 transporter
VOCs	Volatile organic compounds
WUEi	Intrinsic water-use efficiency
Ψs	Stem water potential
